# Supplementary figures and images for: Huntingtin recruits KIF1A to transport synaptic vesicle precursors along the mouse axon to support synaptic transmission and motor skill learning
Source: eLife. 2023 Jul 11;12:e81011. doi: 10.7554/eLife.81011 (PMC10365837; doi:10.7554/eLife.81011)

Figure 5-source data 4

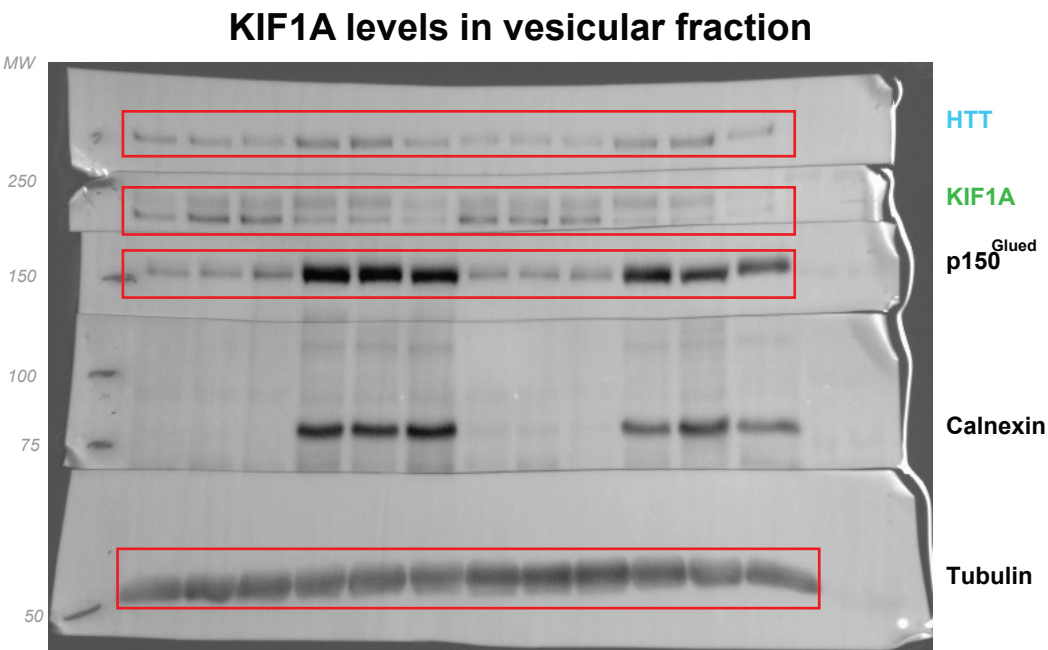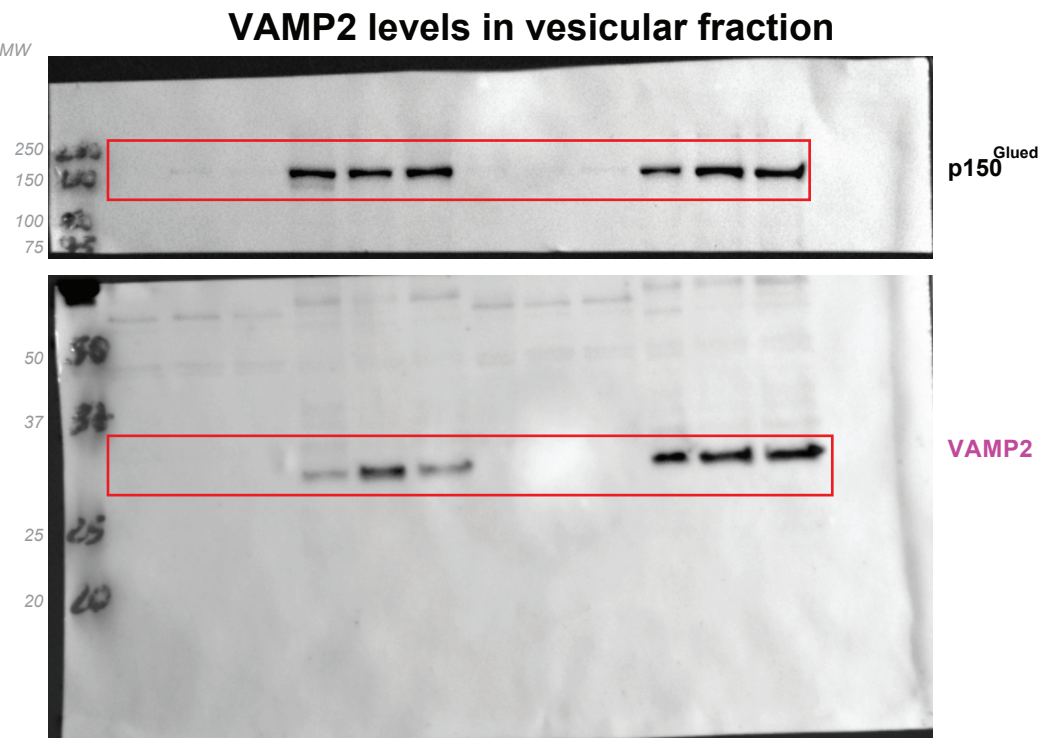

Supplement: Figure 5—source data 4. — Shown in red are the cropped regions presented in Figure 5E. Films containing the second batch of samples (Gel 2) are shown. [file elife-81011-fig5-data4.zip › Figure 5-source data 4.pdf]

Figure 5-Figure supplement 1-source data 6

KIF1A and VAMP2 levels in total fraction

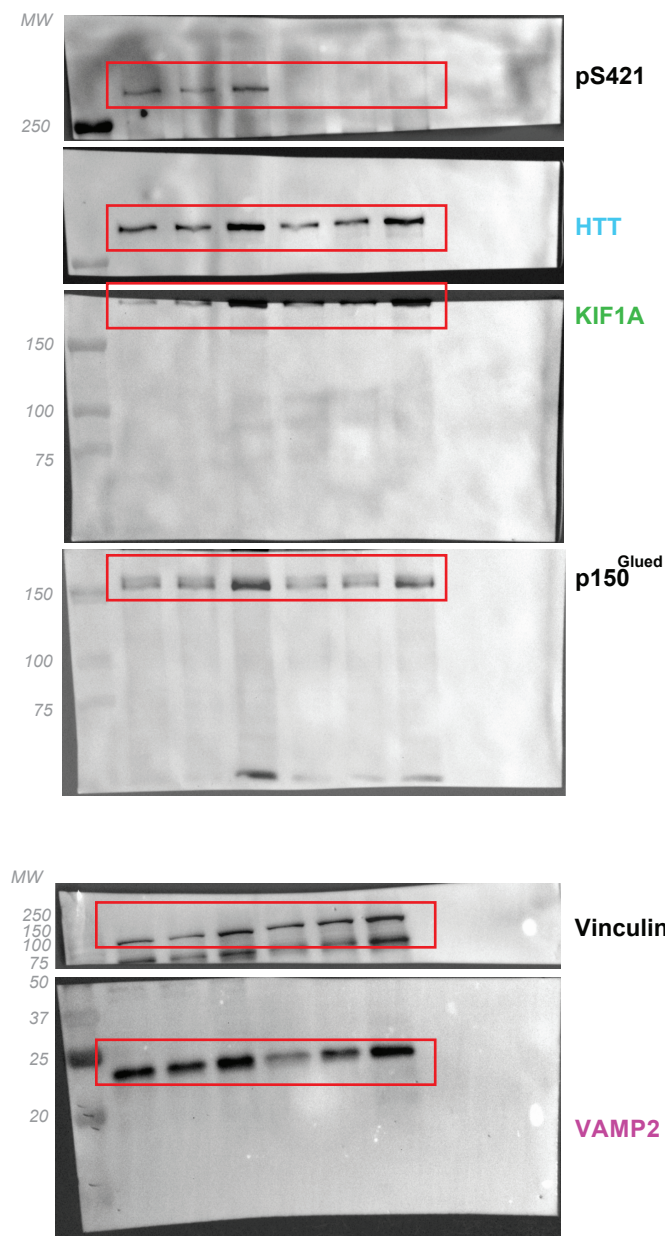

Supplement: Figure 5—figure supplement 1—source data 6. — Shown in red are the cropped regions presented in Figure 5—figure supplement 1D. Films containing the first batch of samples (Gel 1) are shown. [file elife-81011-fig5-figsupp1-data6.zip › Figure 5-Figure supplement 1-source data 6.pdf]
