# Supplementary material for: Huntingtin recruits KIF1A to transport synaptic vesicle precursors along the mouse axon to support synaptic transmission and motor skill learning
Source: eLife. 2023 Jul 11;12:e81011. doi: 10.7554/eLife.81011 (PMC10365837; doi:10.7554/eLife.81011)

Figure 6-Figure supplement 1-source data 1

KIF1A levels in wild-type cortical neurons

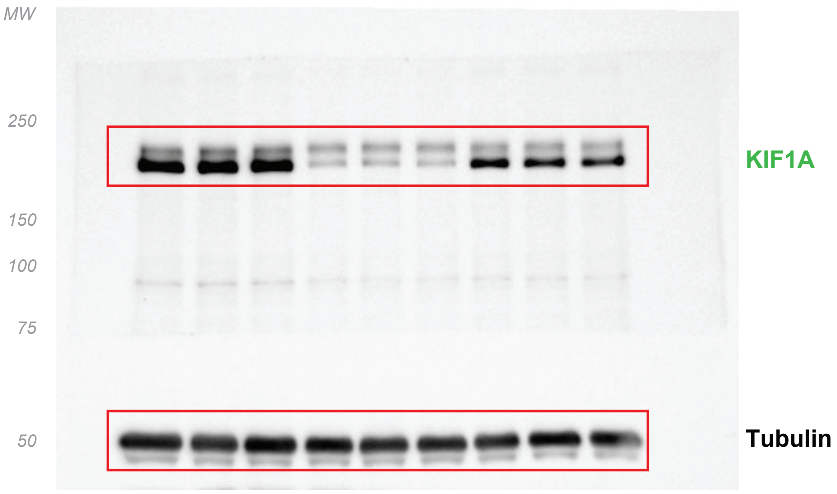

Supplement: Figure 6—figure supplement 1—source data 1. — Shown in red are the cropped regions presented in Figure 6—figure supplement 1A. Films containing the samples are shown. [file elife-81011-fig6-figsupp1-data1.zip › Figure 6Figure supplement 1source data 1.pdf.zip!/Figure 6-Figure supplement 1-source data 1.pdf]
